# Toronto General Hospital

**Published:** 1895-09-14

**Authors:** 


### THE INSTITUTIONAL WORKSHOP.

### HOSPITAL ADMINISTRATION.

### TORONTO GENERAL HOSPITAL.

The General Hospital at Toronto is, on the outside. a handsome building, but, as a matter of fact, it is less good than it looks. It is an old building—comparatively old at least, dating from 1854—and when it was enlarged in 1878 the wings then added did more for the appearance than for the comfort of the hospital. Along each wing a corridor runs, dividing it into two wards. One of these is bright—bright as a strong Canadian sun can make it; the other is dark and chill. But while we cannot praise the arrangement of the building itself, it is possible to speak with entire commendation of the management, and of the special hospitals which exist under the wing and within the *enceinte* of the General Hospital—the Gynæcological Pavilion, the Burnside Lying-in Hospital, and the Nurses' Home. These are all separate buildings within the hospital grounds. The Pavilion dates from 1882; it is a clean-looking structure of white brick, well ventilated, and brought up to modern ideas, and the Lying-in Hospital is practically a separate institution, the occupants of which have no association with the General Hospital while they are there. The Nurses' Home is at the west end of the hospital buildings. It is pleasant, light, and airy, with a comfortable sitting-room; less handsome, indeed, than that in the Victoria Hospital in Montreal, but as pretty and homelike as one need wish.

Like most Canadian hospitals, that at Toronto is not a voluntary hospital. It possesses some property, vested in trustees, but its income is derived chiefly from patients' payments, not merely what we term private patients, though these pay more than others, in consequence of having well-furnished private rooms and sometimes daintier food. But the hospital is subsidised by the province of Ontario, and the county of York, in which Toronto stands, and the city of Toronto itself and other municipalities pay for the patients whom they send there, and the subscriptions, donations, and bequests form an altogether insignificant part of the hospital income. That we think this is to be regretted goes without saying. Bad as it is for our hospitals to be always begging, and almost always in debt, it is, we think, worse that they should drop out of the public remembrance, as State-supported hospitals generally do. But the Toronto Hospital is well managed under the able direction of Dr. O'Reilly, and, under the control of Miss Snively, the matron, a graduate of the Bellevue Hospital, New York, the nurse-training school, founded in 1881, is doing good work in the hospital world.

We think, however, that too much is attempted in too short a time. The training lasts only two years, and includes not only the ordinary run of medical and surgical work, but obstetric and gynæcological nursing. Allowing for the time it takes for the ordinary probationer to grasp the routine of hospital duty, the importance of details that seem trivial to outsiders, allowing, too, for holidays and possible sickness, we think that a longer time is necessary to teach every variety of special nursing. But doubtless the authorities find it difficult to persuade young women to give a longer time to training for their work—'tis a weakness of female human nature to grudge the needful probation time—and give them all they can, crowding instruction into the two years rather than allowing their graduates to go out wholly ignorant of any part of their work. Yet we regret that, since the graduates of Toronto General Hospital go far and wide over the American continent, and in many cases have won positions of honour and responsibility, the training, which lacks nothing of theoretical completeness, should not be prolonged till practical completeness is gained.

From the nurse's point of view there is much to be said for training in a colonial hospital. Not only is the time of training as short as may be, but the probationer is paid from the day she is accepted as a student. The rate of payment in Toronto is, for the first year, three dollars (12s. 6d.) a month, and for the second year six dollars (£1 5s.), while every nurse who completes the course, and passes the final examination with credit, receives, if her conduct during her course has been satisfactory, an additional gratuity of twenty-five dollars (five guineas).

The present writer, when visiting some Canadian hospitals last year, was struck with the fact that the number of nurses often seemed to be large in proportion to the number of beds, but further inquiry usually showed that the nurses were liable to be sent out to private cases at any moment, and that the staff occupied in the hospital itself at any given time was too small rather than too large. Now, while it is permissible and convenient to have a nursing institution in connection with a hospital, where nurses who have completed their training can live, and to which families needing the help of a sick nurse can have recourse, we think that no pupil nurse should be sent out to a private case. That a woman is a good nurse in hospital, with a ward sister or graduate charge nurse to supervise her, and a resident physician within call, does not prove that she is fit for the comparative independence and onerous responsibility of private nursing. Especially with so short a training as two years, we do not see how time is to be found for private nursing. We can understand a nurse being allowed, towards the end of a three years' training, to try her wings in an experimental flight, but in a two years' course we do not see how such a thing can be managed without the omission of something in the way of direct instruction or practice under immediate supervision and guidance.

In our notes of Toronto General Hospital we find an entry that nurses are not sent from the wards to private work. But in the report is a paragraph which seems to contradict this: "Every nurse will be expected to perform any duty assigned her, either as a nurse in the hospital, or when sent to private cases among the rich or poor in any part of the province." As the staff is not by any means excessive—60 nurses to 400 beds—we do not see how any can be spared without the efficiency of the hospital suffering. Perhaps there is a reserve corps of nurses who can be made available for private work, and we by no means deny that here and there a nurse may be found of such capacity as to make her, quite early in her career, fit for difficult and onerous work, but as a general rule the incompletely trained private nurse is as much to be condemned as the unqualified practitioner.

The fees charged for private nurses, ten dollars (two guineas) a week, and in contagious cases two dollars a day (£2 18s. a week) doubtless form an appreciable item in the income of the training school, but if possible it would be better to reduce expenses by withdrawing or lowering the payment for the first year of training, rather than to add to income by sending pupil nurses to private cases. While it is difficult to criticise and rash to condemn the methods of work in vogue in a country where the conditions of life are different from our own, we think that the prominent position held by the Toronto General Hospital, and the importance of the work of its training school for nurses, should make it aim at giving its graduates the best and most complete equipment for duties which in a country so large and so thinly peopled as the Dominion of Canada are even more responsible than they are in Britain.

